# The ways parents cope with stress in difficult parenting situations: the structural equation modeling approach

**DOI:** 10.7717/peerj.3384

**Published:** 2017-06-12

**Authors:** Agnieszka Szymańska, Kamila Anna Dobrenko

**Affiliations:** 1 Institute of Psychology, Cardinal Stefan Wyszynski University in Warsaw, Poland; 2 Institute of Psychology, The Maria Grzegorzewska University, Warsaw, Poland

**Keywords:** Discrepancy, Reactions to stress, Parental difficulty, Representation of the child

## Abstract

The purpose of this study was to verify a theoretical model of parents’ responses to difficulties they experienced with their child. The model presents relationships between seven variables: (a) discrepancy between parental goal and the child’s current level of development, (b) parental experience of a difficulty, (c) representation of the child in the parent’s mind, (d) parent’s withdrawal from the parenting situation, (e) seeking help, (f) distancing oneself from the situation, and (g) applying pressure on the child. The study involved 319 parents of preschool children: 66 parents of three-year-olds, 85 parents of four-year-olds, 99 parents of five-year-olds and 69 parents of six-year-old children. Structural equations modeling (SEM) was used to verify the compounds described in the theoretical model. The studies revealed that when a parent is experiencing difficulties, the probability increases that the parent will have one of two reactions towards that type of stress: withdrawal from the situation or applying pressure on the child. Experiencing difficulties has no connection with searching for help and is negatively related to distancing oneself from the situation.

## Introduction

Partental experience of difficulty in a parenting situation is understood as the internal state of tension caused by a *difficult situation* (Gurycka, 1979). Coping with difficulties is a typical stress-related state. Therefore, experiencing difficulty is treated in the literature as a synonym of mental stress (Reykowski, 1966). The term *experiencing difficulty in a parenting situation* narrows down the scope of the concept to situations related to interaction with the child, i.e., situations that are characteristic of the parenting process. In other words, experiencing difficulty in the parenting situation is synonymous with experiencing stress in that situation. A stressful situation must be coped with and a way must be found to reduce the experienced stress. In addition to the two most fundamental reactions to stress, i.e., flight (withdrawal from the stressful situation) or fight (Selye, 1956), two more responses are possible, i.e., cognitive distancing oneself from the stressful situation or seeking help. Many factors determine people’s responses to stress. These include: representation of the stressor (Eschleman Kevin et al., 2012), personality traits (Higgins & Hughes, 2012), motivation to cope with the stress and chronic stress (Ebstrup et al., 2011), stress intensity and perceived social support (Howard & Hughes, 2012), representation of oneself as a person who can cope with stress, etc. But the way one copes with stress can also affect one’s goal achievement (Gaudreau, Carraro & Miranda, 2012).

In the present study we present a new, never previously tested model which describes how parents cope with stress and the reasons for parental stress. The model tests four possible reactions to stress: (a) withdrawal, (b) defense via application of pressure, (c) seeking help and (d) cognitive distancing oneself from the stressful situation. The variable mediating between experienced difficulty and the stress reaction was parental mental representation of the child. The first variable in the model which initiates the entire phenomenon is discrepancy between parental goals (features that parents want their child to develop) and the child’s state (the extent to which the child has developed those features). This discrepancy is treated as the cause of parental stress and leads to the situation in which the parent has to cope with difficulties.

## Theoretical Context

Gurycka’s theory of parental mistakes (Gurycka, 1990; Gurycka, 2008) provides the theoretical framework for the study of the influence of experiencing difficulties in the parenting situation on one’s reaction to stress. The model presented here was reconstructed on the basis of Gurycka’s theory (Gurycka, 1990; Jonkisz, 1998; Szymańska, 2016).

### Association of parenting discrepancy with the difficulty experienced by the parent

Parenting **discrepancy** (Gurycka, 1979), a concept used in educational psychology, is defined as the distance between parental goals, i.e., the mental characteristics that the parent wants the child to develop in the course of the educational process (Muszyński, 1972; Sośnicki, 1966) and the current state of the child, i.e., the degree to which the child has acquired such characteristics (Gurycka, 1979). This discrepancy is the result of observation of the differences and its nature is purely cognitive.

According to Gurycka (1979), parenting discrepancy is the primary variable from which analysis of each of the parenting processes should begin. *The validity of this principle when determining the parenting process is explained by the fact that often, depending on the magnitude of this discrepancy, the child needs to be provided with specific experiences to achieve the chosen goal* (Gurycka, 1979). Discrepancy between the current and desired states evokes motivational states (Kochańska, 1982). When obstacles which prevent or delay achievement of the objective appear on the way to achieving the objective, they produce emotions of anxiety, irritation and despair (Reeve, 2005).

According to Gurycka (1990), the greater the distance between the desired characteristics and the current characteristics of the child, the greater the perceived difficulty (stress). Parenting difficulty as experienced by the parent is an internal state characterized by tension caused by the difficult situation that can make the parent need to reduce the discrepancy (Reykowski, 1966; Gurycka, 1979; Kochańska, 1982; Reeve, 2005). It is a reaction to the discrepancy at an emotional-motivational level.

Experiencing difficulty therefore carries the message that the concept is the same as the concept of mental stress, i.e., it refers to difficult situations (Reykowski, 1966). This distinguishes the experienced difficulties from the experience of positive stress when it appears as a result of a positive experience, e.g., marriage, the birth of a child, etc. (Ledzińska, 2009). Addition of the adjective *parenting* to the notion of difficulty or its relocation to *the parenting situation* limits its scope only to situations related to the child-raising process. These situations are internal experiences of the parent characterized by tension and related to parenting situations which refer only to the process of raising and interacting with a child.

Difficulty as experienced by the parent is combined in the model with task solving (Gurycka, 1990). It is understood that the difficulty is related to the effort the parent puts into the parenting process. Experiencing difficulties is thus the result of the inability to achieve the planned parental goal because the parent is unfit, unprepared, or he/she has to make a very great effort in order to reconcile the discrepancies. The purpose is to provide the child with experiences enabling reduction of the discrepancies between parental goals and the child’s current state (Gurycka, 1979).

In the case of a discrepancy, parental motivation increases and the power of the routing process runs as follows: parental control, conversations with the child and so on. If the parent fails, he/she might experience difficulties and psychological stress, which that parent will then have to deal with. The parent may therefore experience difficulties (stress) as the effect of the inability to achieve parental goals.

In conclusion, there are theoretical reasons to believe that the discrepancy between parental goals (parenting objectives) and the child’s actual level of development may be a determinant of experienced parenting difficulties. This is the content of the first hypothesis in the proposed model (H1).

### Connection between difficulties experienced by the parent and the representation of the child in the parent’s mind

The representation of the child in the parent’s mind is formed as a result of experiencing difficulties. Representation of the object is formed in the course of interaction with it (Valentino et al., 2008; Zeanah, Mammen & Lieberman, 1993) or is upgraded as a result of those interactions (Szymańska, 2012a). The parent may shape his/her representation of the child as a result of experiencing difficulties in the parenting situation. The association of experiencing difficulties with the representations of the child in the parent’s mind constitutes the content of the second research hypothesis (H2).

A closer look at the notion of representation itself should be taken in order to analyze the role of the child’s representation in the parent’s mind and its impact on parenting attitudes. Representation in psychological terms is: a thing which means something, occupies the place of something or symbolizes something (Reber & Reber, 2005). In developmental psychology, the concept of representation was introduced by Piaget. Different meanings of this term appeared when cognitive concepts were introduced. Our understanding of the concept of **representation** is taken from Gurycka (1994), who defined it as dynamic, subjective cognitive patterns which are shaped by experiences and at the same time are subject to change as a result of these experiences. At the core of the formation of the representation of the world in the human mind is experience (Gurycka, 1996).

Other authors wrote similarly about this aspect of representation and claimed that representation is a memory structure reflecting the version of life experiences (Zeanah, Mammen & Lieberman, 1993; Valentino et al., 2008; Greenberg, 2002).

Representation is therefore an image, a memory of a person’s former experience which describes the human view on reality (Gurycka, 1994; Gurycka, 1996). Representation of the child in the mind of the parent reflects the image of the child in the mind of the parent, which is formed on the basis of the experience of interacting with that child and is sometimes updated on the basis of this experience (Gurycka, 1996).

Pratt & Gaston (1993) mention two types of representation: representation by something and representation of something. Representation by something uses mental symbols to represent reality. Representation of something refers to the object of representation (e.g., representation of the child) (Pratt & Gaston, 1993). A parent describing the child relates to his/her representation of that child as stored in his/her mind.

When writing about experience as a factor implying the formation of representation in the mind of a parent, Gurycka (1990) presents a causal relation between the discrepancy, experienced difficulty and the representation of the child.

The impact of the experience on the development of the representation of the child in the parent’s mind is the second research hypothesis of this model (H2). As a result of the experienced difficulties, in the mind of the parent there appears a specific representation of the child and of his/her activity as less important than the activity of the parent. Such a depreciation of the child’s activity may result from the parent’s internal need to justify the fact that he/she prefers to follow his/her own goals instead of the needs and activities of the child (this is one of the many possible representations of the child that can be formed in difficult situations).

In the proposed model, the representation of the child acts as a mediator between the difficulty experienced by the parent and the parent’s reaction to the stress that is used.

### Connection between the representation of the child in the parent’s mind and the reaction to stress

Gurycka also wrote about representation in the context of the theory of psychological stress (she referred to Reykowski’s concept of stress). In light of Reykowski’s (1966) theory, a difficult situation is connected with shaping the representation of the entire event and the people involved. Among later theories of stress, a similar approach was suggested by Lazarus et al. (1985). In his theory, Reykowski stated that a person’s reaction to a difficult situation depends on how the person perceives the situation. Therefore, the parental reaction to a difficult situation will depend on the shape of the representation of the child in the parent’s mind.

According to Reykowski’s (1966) theory of psychological stress, the parent in this situation may adopt one of two strategies: fight the stress or select a defense strategy against that stress. The inspiration for this division of reactions was biological theories describing the stress response of *fight or flight* (Selye, 1956). The choice of reaction to stressful situations may depend on the individual’s temperament or personality (Strelau, 2002), as well as on factors such as the ability to influence the situation that causes stress. Gurycka (1990) writes that *confused or upset by difficulty, a parent who works under stress tries to defend him or herself or succumbs*.

Folkman & Lazarus (1980) and Lazarus et al. (1985) described coping with stress as constantly changing the cognitive and behavioral efforts aimed at mastering certain external and internal requirements which are evaluated by a person as aggravating or exceeding his/her resources (Chen et al., 2009; Folkman et al., 1987; Wrześniewski & Guzowska, 2002). The strategies described in this context are: positive revaluation, focusing on the problem, and creating positive events (important for survival and development) (Bandura, 2005; Folkman & Lazarus, 1980; Krypel & Henderson-King, 2010).

Hobfoll stated that differences in coping with stress mainly result from the fact that people have different resources (the resources are not equal). One of the strategies of coping with stress is to re-interpret the threat as a challenge and to focus on what one can earn in a given situation (i.e., what resources can be gained) (Hobfoll & Lerman, 1989; Hobfoll, 2006; Hobfoll & Lilly, 1993; Hobfoll, 2011).

Endler & Parker (1990a) describe three styles of coping with stress. The task-oriented coping involves making an effort to solve the problem and to change the difficult situation. The emotion-oriented coping manifests itself mainly in one’s focusing on emotions. The avoidance-oriented coping involves focusing on alternative activities rather than on the problem. This division was included in the measurement tool of the Coping Inventory for Stressful Situations—CISS (Endler & Parker, 1990a; Livneh et al., 1996).

Reykowski’s stress response model takes into account both adaptive and maladaptive responses to stress and the reactions of fight and flight (Reykowski, 1966; Terelak, 2008). On this basis, four categories concerning stress response were studied in the proposed model. These reactions and their examples, as described by Reykowski and applied by Gurycka in her theory, are presented in Table 1.

**Table 1:** Four categories of stress response by Reykowski.

	Adaptive	Maladaptive
Fight	Cognitive distancing	Pressure
Flight	Help seeking	Withdrawal

**Withdrawal** as a reaction of defense against stress (which is a maladaptive reaction). It is based on withdrawal from the difficult situation.

**Getting help** as an adaptive defense against stress. It involves seeking help to find a solution (e.g., consulting the child’s grandparents, a psychologist, etc.).

**Cognitive distancing** (looking for other solutions) as an adaptive response to combat stress. It involves analyzing the situation from a distance which favors change in perspective and acceptance of different solutions.

**Pressure** as a maladaptive reaction to combat stress. It involves the application of pressure in order to fight the difficulties.

This approach to coping with stress has rarely been explored and is therefore worth putting to the empirical test, all the more so that Gurycka’s theory (Gurycka, 1990; Szymańska, 2012a) refers precisely to this approach to stress.

According to Gurycka, a parent who considers the representation of the child and that child’s tasks as less important than the parent’s tasks can apply pressure to the child in order to interrupt that child’s activity or to subordinate the child to the tasks of that parent. Connections between the representation of the child and combating stress by means of pressure is the content of the third research hypothesis (H3). Yet a parent having such a representation of the child may also react differently and withdraw from the parenting situation altogether. This is the content of the fourth research hypothesis (H4).

The parent might also react to a difficulty in the parenting situation in two different ways: either by asking for help (H5) or by cognitive distancing him or herself from the stressful situation (H6) (Reykowski, 1966). In both adaptive reactions (help-seeking, cognitive distancing) the parent has a chance to review his/her representation of the child and to rethink his/her parenting objectives (Gurycka, 1990).

## Limitation of the Model Reconstructed on the Basis of Gurycka’s Theory

Gurycka’s model contains many more psychological components than the ones mentioned here, e.g., parental personality, the child’s temperament, parental values, all of which can affect the choice of parenting goals. According to Gurycka (1990) these variables can either determine the choice of parenting goals directly or at least mediate the relation between discrepancy (the parenting goals assumed by the parent versus the level of development of a particular trait in the child) and parental experience of difficulty in the educational situation.

Research on parental difficulties and parental stress has demonstrated that a child’s temperament can be a variable which moderates many parental behaviors (Casanueva et al., 2010; Lee, 2013; Puura et al., 2013; Phillips et al., 2012). It may also determine the way in which a parent experiences interactions with the child and determine the level of stress that is experienced by parents and parental welfare (Bruning & Mcmahon, 2009; Casalin et al., 2014; Laukkanen & Ojansuu, 2014; Mclean, 2012; Oddi, Murdock & Vadnais, 2013).

The presented model, reconstructed on the basis of Gurycka’s theory, does not include these constructs at this stage of analysis because this would make the model so complex that it would be untestable. Exclusion of parental personality traits and the child’s temperament limits the present model. These variables can be tested in future research and analyses.

Also, discrepancy as the main determinant of parental experience of difficulty, postulated by Gurycka, is just one of many possible variables explaining difficult parenting situations and parental responses to stress. Other possible psychological determinants of parental difficulty have also been postulated in the literature, e.g., such difficult child behaviors as external and internal behavioral problems (Szymańska, 2017). According to this research approach parents experience difficulty because “the child is difficult”. In these models the child’s behavior is viewed as the cause of parental difficulty. In those studies where it is assumed that parental difficulty is caused/explained by the child’s difficult behavior researchers also try to identify parental behaviors which can cause these difficult child behaviors, e.g., parent–child communication styles (Szymańska, 2012b).

Another research approach to difficulties seeks the causes of these difficulties in the way the child is represented in the parent’s mind. Bugental & Shennum (1984) found that the mental representation of the child as “well-behaved” versus “difficult” significantly affected parental communication with the child. Bugental et al. (1999) found that parents who had a more negative mental representation of the child (“difficult child”) were more verbally aggressive toward the child. She also found that parent’s self-confidence had a very powerful effect on their behavior toward the child. Parents with low parental self-confidence behaved less adequately (Bugental et al., 1999). As these examples show, theories endorsed by other researchers suggest that variables other than those postulated by Gurycka may determine parents’ experience of difficulty and their behavior in difficult situations. Both Gurycka’s theory and Bugental’s research have demonstrated that parents’ mental representations of the child have a significant effect on their behavior toward the child.

Many variables not postulated in Gurycka’s theory or the model reconstructed on the basis of her theory may cause parents to experience difficulty. It is worth noting, however, that what distinguishes the present model from other models presented in the literature is that it locates the causes of difficulty in failure to achieve goals. It therefore has an affinity to stress theory.

Gurycka’s constructs of discrepancy, experienced difficulty and representation clearly fit into stress research. In this approach considerable importance is attributed to failure to achieve goals as a determinant of experienced difficulty (stress) (Reeve, 2005). Experienced difficulty affects the representation of the object which is causing stress, finally leading (depending on the type of representation) to specific coping responses (Reykowski, 1966). When developing her theory, Gurycka referred to stress theory and to Tomaszewski’s  (1975) theory of difficult situations and theory of situations of deprivation.

The model reconstructed on the basis of Gurycka’s theory is one of many models explaining the origins of parenting difficulties. The purpose of the present study was to test the adequacy of the model reconstructed on the basis of Gurycka’s theory. If this model can be successfully tested empirically, i.e., its rejection will be unfounded and the relations within the model will be powerful; this will mean that stress explains parents’ behavior toward their children, thus corroborating many existing studies. The mechanism underlying the origin of the difficult situation (stress), i.e., failure to attain goals, may explain the experience of difficulty in relations with the child. This would be an important and novel contribution of Gurycka’s theory to the development of science.

## Theoretical Model and Hypotheses

**Figure 1:**
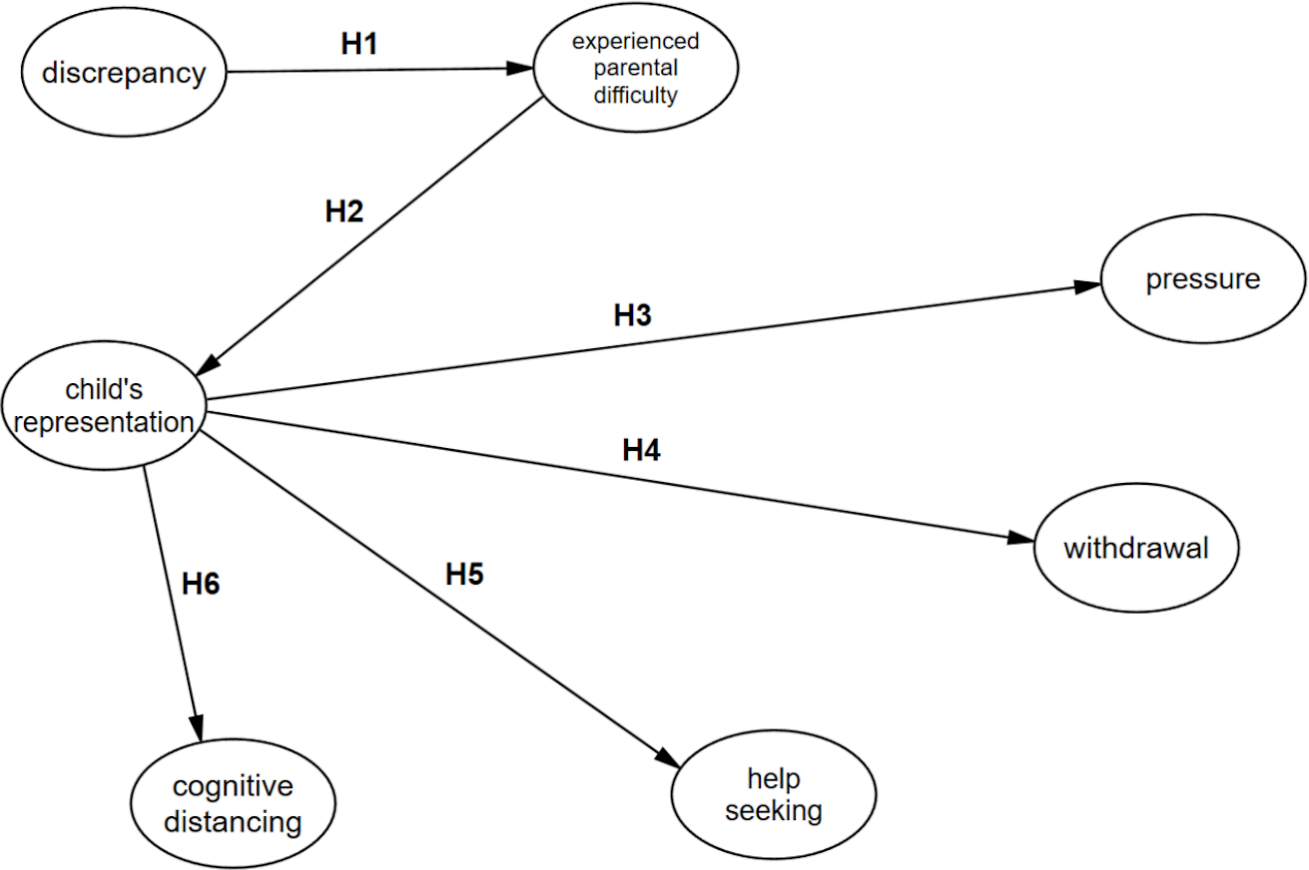
Substantive model (theoretical) for the relationships checked here. The letter H indicates relationships between concepts which are later described as the research hypotheses.

The purpose of the present study was to test this model (Fig. 1).

The following hypotheses were put forward:

**H1:** Discrepancy is positively correlated with the difficulty experienced by parents in the parenting situation—this is a hypothesis put forth explicitly by Gurycka (1979) in her work.

This means that the larger the discrepancy between the objectives assumed by the parent (the psychological traits the parent wants the child to develop) and the child’s current level of development, the greater the difficulties the parent experiences in the relationship with the child.

**H2:** Difficulty (stress) experienced by the parent in the parenting situation correlates positively with a negative representation of the child and its tasks in the parent’s mind–this hypothesis was put forth explicitly by Gurycka (1996) in her works.

The representation of the child is formed in the course of interaction with that child as a result of experiences in the relationship with the child, but it may also result from the general perception of the role of the parent and the child.

**H3:** The representation of the child correlates positively with combating stress by applying pressure. This hypothesis was put forth *explicitly* by Gurycka (1990).

The aim of applying pressure by the parent is to force the child to surrender either by coercion or by punishment.

**H4:** The representation of the child in the parent’s mind correlates positively with the parent’s reaction as defense against stress by withdrawal. This hypothesis was put forth *implicitly* by Gurycka (1990) in her work in reference to the concept of mental stress.

**H5:** The representation of the child in the parent’s mind is related to the response to combat stress by seeking help. This hypothesis was put forth *implicitly* by Gurycka (1990) in her work in reference to the concept of mental stress.

**H6:** The representation of the child in the parent’s mind is related to parental combating of the stress reaction by distancing oneself from the situation. This hypothesis was put forth *implicitly* by Gurycka (1990) in her work in reference to the concept of mental stress.

## Method

### Ethics committee approval

*All procedures performed in studies involving human participants were in accordance with the ethical standards of the institutional and/or national research committee and with the 1964 Helsinki declaration and its later amendments or comparable ethical standards*. The research study received the approval of the Ethics Committee of the Faculty of Psychology, Warsaw University on January 12, 2010.

### Participants and procedure

The study was conducted online on the territory of Poland. The questionnaire were posted on a website for parents. The study sample was random. The sampling frame was a list of preschools provided by the Ministry of Education in Poland. The interval draw was constant *k* = 6. Preschools representing all voivodeships and provinces in Poland were drawn (group draw), proportionally for the largest provinces. Randomly selected preschools were contacted via email, and preschool directors were asked to inform parents about the research. A large portion of people invited to the study refused to participate. Those who did agree to take part in the study acquainted themselves with detailed information on how the testing would be done on the website.

At the beginning of the study the parents were asked to think about their child, i.e., the one that was attending preschool, and to answer questions pertaining only to that child until the end of the study. This procedure protected against a criss-cross of responses if the parent had more than one child. The study involved 319 participants–both the fathers and mothers of the preschoolers.

The participants’ ages ranged from 19 to 54 years (the group can be described as a group of young adults); the mode was 33 years, the median was 27 years. It can be said that the study group was dominated by well-educated people. The mode indicates that the biggest group was the group of people with a university education (63.4% of the sample); 4.6% of the sample had primary and vocational education; 29% had secondary education; 2.9% had PhD.

The subjects were mainly from big cities (city population of 50,000–200,000, 22.3% of sample; city population of 200,000–500,000 11.4% of sample; city population of >500,000, 26.3% of sample), but the study also included subjects from rural areas and small towns (rural 13.1% of sample; town population up to 10,000 6.9% of sample; town population of 15,000 20% of sample).

In the sample was an overwhelming number of mothers (90.3%) over fathers (9.7%). The study sample included a comparable number of parents of both boys (175, 54.9%) and girls (144, 45.1%).

The study included parents of preschoolers aged 3–4 years (151 children, 47.3% of sample) and 5–6 years (168, 52.7% of sample). The distribution of the children’s gender in the different age groups was as follows: in 3 year old group number of girls was 34 (51.5% of sample), boys 32 (48.5% of sample); in 4 year old group number of girls was 34 (40% of sample), boys 51 (60% of sample); in 5 year old group number of girls was 52 (52.5% of sample), boys 47 (47.5% of sample); in 6 year old group number of girls was 24 (65.2% of sample), boys 45 (34.8% of sample).

The children of the parents taking part in the study attended state-run (186 children, 58.3% of sample) as well as private (67 children, 21% of sample), Catholic and other preschools (58 children, 18.2% of sample). The largest number of children attended state-run and private preschools, fewer children went to Catholic preschools and the smallest number of children attended Montessori preschools.^1^ 1However, this is the rarest type of preschool in Poland.

The research sample has many characteristics of a representative sample. It is almost equal in number for the children’s gender (the approximate number of boys and girls born in Poland). We also attempted to reflect the frequency of preschools (there are more public preschools in Poland). In Poland, 60% of people live in cities and 40% live in rural areas. Only 13% of our sample came from rural areas. However, it should be remembered that the study involved the parents of children attending preschool. Few children from rural areas in Poland attend preschool, therefore this category may be underrepresented. The level of education of the respondents is not surprising, since as many as 50% of women in Poland (aged 30–34 years) have higher education. In our sample 63% had higher education. This discrepancy may be due to the specific nature of the sample. The sample of fathers (men) was very underrepresented in our sample. There could be various reasons why mothers, and not fathers, agreed to take part in the research. One possibility, culturally conditioned, is that in Poland young children are looked after more often by women.

### Study plan

Tha study was correlational. The variables were as follows:

**Discrepancy** is conceptualized as the distance between parenting objectives (the psychological traits which the parent wants to shape in the child in the course of the parenting process) (Brzezińska, 2002; Glenn, 2005; Gurycka, 1979; Miller, 1966; Muszyński, 1972; Szymańska, 2011a; Szymańska, 2012a) and the child’s current state, i.e., the degree to which the child has developed a desired characteristic (Gurycka, 1979). Discrepancy is the result of the observation of differences and has a purely cognitive character. It was measured by means of the Discrepancy scale.

**Experiencing difficulties in the parenting situation by a parent** is the internal state of the parent characterized by tension caused by a *difficult situation* which motivates the parent to reduce the discrepancy (Gurycka, 1979; Kochańska, 1982; Reeve, 2005; Reykowski, 1966). It is a reaction to the discrepancy at the emotional-motivational level. It was measured by means of the Experienced Parenting Difficulties scale.

**Representation of the child in the parent’s mind**—*the memory structures that represent the version of the life experiences of an individual* (Valentino et al., 2008; Zeanah, Mammen & Lieberman, 1993)—reflect the image of the child in the parent’s mind, which is formed on the basis of the experience of interaction with the child or is sometimes updated on the basis of these experiences. This paper examines the representation of the child and the child’s tasks *as tasks that are unimportant or at least less important than the ideas, objectives and needs of the parent* (Gurycka, 2008). It was measured by means of the Representation of the Child in the Parent’s Mind scale.

**Reaction to stress**—can assume four forms:

(a) combating the stress reaction by **using pressure**, which is focused on achieving objectives despite anything and by overcoming obstacles such as, for example, the child’s resistance. Reactions of combating stress by using pressure take place along with mobilization (excitement, increase in the intensity of the reaction, acceleration of the pace and fluidity of associations, etc.).
(b) **cognitive distancing** from a stressful situation by examining that situation and modifying the course of actions.
(c) **seeking or requesting help**.
(d) defense against stress by removing oneself from the stressful situation, symbolic **withdrawal**, seeking refuge (Reykowski, 1966). Defense reactions to stress are an expression of destruction and therefore bring disorder to the organization of activities and self-control, a sign of inability to build an action plan and to make reasonable decisions in order to accomplish objectives (Reykowski, 1966). They serve to protect individuals against the harmful effects of stress by their moving away and resigning from achieving the objectives. This was measured by means of the Reaction to Stress scale.

### Measurement tools

Since no psychological instruments measuring the tested variables exist it was necessary to construct such instruments. The instruments and their psychometric properties are described below. All items in the scales that we constructed were included in the tested model. No item was rejected. Four measurement tools were constructed.

#### Discrepancy scale

In order to measure the parents’ parenting objectives we used a scale which is designed to measure the parent’s parenting goals (mental traits that the parent wants the child to develop) and the distance between the parenting goals and the child’s current level of development of these characteristics (Szymańska, 2011a; Szymańska, 2012a).

The scale consists of 12 questions which are arranged in pairs. Each pair of questions measures the parent’s response concerning one parenting objective. The first question in each pair refers to the parenting objective. Parents are asked to list the qualities they would like to form in the child (see Table 2). At the same time, on a scale from −7 to 7, they are to estimate how much they want the child to develop the given feature. The second question concerns the extent to which the child has a particular trait developed at the present moment. Parents, on a scale from −7 to 7, estimate the degree of ownership of this feature by the child.

**Table 2:** First pair of questions in the Discrepancy Scale of parental goals.

INSTRUCTIONS
Please list three traits that are especially important to you as a parent and which you make an effort to make sure your child develops. **Trait one:** (enter trait name here) Mark how important this trait is to you as a parent and the extent to which you wish your child were like this. - 7 - 6 - 5 - 4 - 3 - 2 - 1 0 1 2 3 4 5 6 7 (- 7) definitely not like this (7) definitely like this Mark the extent to which (write your child’s name here) has developed the trait in question. - 7 - 6 - 5 - 4 - 3 - 2 - 1 0 1 2 3 4 5 6 7 (- 7) definitely has not (7) definitely has

Three pairs of questions in the scale relate to positive traits (traits that the parent would like to see developed in the child); these are followed by three pairs of negative traits (traits that are unwanted by the parent).

The distance of the parenting objective to the child’s current state is measured by the square of Euclidean distance.^2^ 2The results of the first question are subtracted from the results of the second question in the same pair and the difference is squared.This way six measurements are achieved (three on the desired objectives and three on the undesired ones).

**Validity.** The results of the exploratory factor analysis EFA confirmed the existence of two factors. The first factor explaining 30.449% of the variability of all the results measures the discrepancy from the undesired traits (items: disc4–disc6). The second factor explaining 29.873% of the variability measures the discrepancy from the characteristics desired by the parent (items disc1–disc3).

**Reliability.** The scale has high reliability. The discrepancy from the positive objectives has a *α* − *Cronbach*′*s* = .850, RO2 = .653; the discrepancy from the negative objectives has a *α* − *Cronbach*′*s* = .806, *RO*2 = .581.

#### Experienced parental difficulty scale

The scale of Experienced Difficulty in the Parenting Process (scale DTW) measures the level (severity) of difficulties experienced by the parent in his/her the relationship with the child.

**Validity.** Exploratory factor analysis EFA (Varimax method) showed the existence of a single factor explaining, altogether, 74.966% of the variance of all results.

**Reliability.** Internal consistency Cronbach’s alpha was .965. Reliability calculated by intraclass correlation RO2 = .773.

#### Stress scale

The Stress Scale comprises 15 items measuring the parent’s response to stress in the parenting situation.

**Validity.** Exploratory factor analysis EFA revealed the existence of four factors, explaining altogether 68.910% of the variability of all the results. The first factor (explaining 24.270% of the variability) is a factor related to defense against stress. It includes items revealing that the parent withdraws from the parenting situation. The second factor (explaining 16.435% of the variability) is a factor related to combating stress by trying to overcome obstacles (pressure application). The third factor (explaining 15.520% of the variability) is a factor related to combating stress by distancing oneself from the parenting situation and forming a positive attitude towards the situation (cognitive distancing). The last, the fourth factor (explaining 12.684% of the variability), is a factor associated with coping with stress by trying to find a way out of the situation (i.e., seeking the help of a specialist).

The Stress Scale was validated with Endler and Parker’s Coping Inventory for Stressful Situations (CISS) (Endler & Parker, 1994; Endler & Parker, 1990b), adapted to Polish by Strelau and collaborators (2005). It was assumed that correlations should be low to moderate because one of the instruments measures the general coping construct (reaction to stress in various situations) whereas the other one measures coping with stress in a specific, upbringing situation. The same person may respond differently in the two tests. It was assumed at the theoretical level that:

(a) the Cognitive Distance scale would correlate positively with Task-Oriented Coping and negatively with Emotion-Oriented Coping;
(b) the Help-Seeking scale would correlate positively with Social Diversion;
(c) the Withdrawal scale would correlate positively with Avoidance-Oriented Coping and Distraction;
(d) no assumptions were made concerning the Pressure scale because none of the CISS subscales replicates this theoretical construct.

The convergent and divergent validity of the Stress Scale were verified by means of Confirmative Factor Analysis and the following results were obtained:

ad. (a) Positive Distance correlated positively with Task-Oriented Coping (.608, *p* < .05) and negatively with Emotion-Oriented Coping (−.413, *p* < .05). These results confirm the convergent and divergent validity of the Cognitive Distance Scale;
ad. (b) the Help-Seeking Scale correlated positively with Social Diversion (.470, *p* < .05) and negatively with Emotion-Oriented Coping (−.527, *p* < .05), confirming the scale’s convergent validity;
ad. (c) the Withdrawal Scale correlated positively with Avoidance-Oriented Coping (.342, *p* < .05) and Distraction (.512, *p* < .05), also confirming the scale’s convergent validity;
ad. (d) no correlations were found between the Pressure Scale and any of the CISS subscales. Pressure should not and does not correlate with any of these coping styles.

**Reliability.** Reliability of the tool was calculated for each scale. Reliability of the withdrawal scale was *α* = .893, RO2 = .583; pressure scale *α* = .917, RO2 = .787; cognitive distancing scale *α* = .904, RO2 = .757; and seeking help scale *α* = .768, RO2 = .525.

### Measurement model

The first step in calculating the SEM model was to construct a measurement model to determine whether the latent variables had been constructed correctly (Bartholomew et al., 2008; Hair et al., 2006; Szymańska, 2016). On the basis of the psychological characteristics, seven latent variables were constructed: (a) discrepancy, (b) difficulty experienced in the parenting situation, (c) representation of the child, (d) reaction of combating stress by applying pressure, (e) defensive withdrawal from the situation, (f) reaction of combating stress by distancing from the situation (cognitive distancing), and (g) defensive help-seeking.

Characteristics of the latent variables:

**Discrepancy**, the latent variable, consists of items belonging to the Discrepancy scale. Lambda (factor loadings) of the observable variables (test items) were, respectively, *λdisc*1 = .864; *λdisc*2 = .820; *λdisc*3 = .755; *λdisc*4 = .694; *λdisc*5 = .804; *λdisc*6 = .796. All items (observable variables) were associated with the latent variable at a significant level. The latent variable consists of two factors. The first factor consists of questions relating to the positive objectives (the traits that the parent would like to develop in the child). This factor includes observable variables: disc1, disc2, disc3. The second factor consists of questions relating to the negative objectives (the traits that the parent would not like to have develop in the child). The second factor includes observable variables: disc4, disc5, disc6.

**Figure 2:**
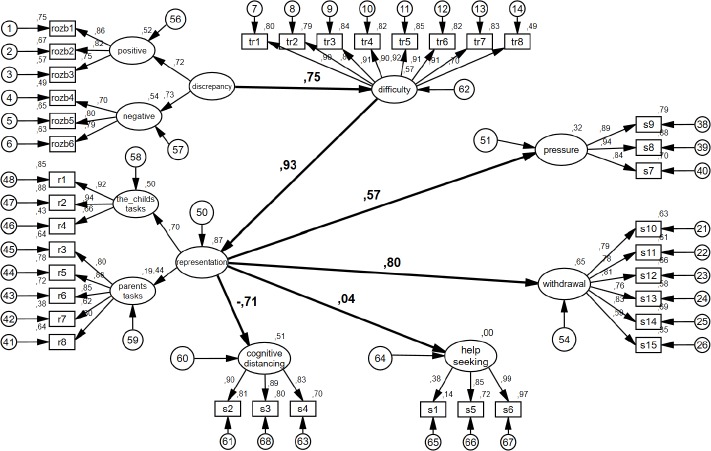
Graphical representation of the model. Standardized results. The relationships between the latent variables are bold.

The variance extracted for the first factor (see Fig. 2 in the *positive* circle) is VE = .663, and reliability CR = .855. Variance extracted for the latent variable of the negative discrepancy (see Fig. 2 in the *negative* circle) is VE = .587, and reliability CR = .809. For the hierarchical variable of the discrepancy, variance extracted on the basis of these two factors was VE = .525, and reliability CR = .689.

**Difficulty in the parenting situation**^3^ 3The latent variable consisted of the following observable variables:dif1 I have many parenting problems with my child.dif2 I have the impression that bringing up my child is a constant struggle.dif3 I experience parenting problems associated with my child.dif4 I am constantly upset due to conflicts with my child.dif5 I often experience powerlessness in contact with my child.dif6 I am constantly angry due to my child’s behavior.dif7 I cannot cope with my child.dif8 I experience a lot of anxiety in contact with my child.consists of items belonging to the scale of Difficulties experienced in the parenting situation. Lambda (factor loadings) of the observable variables (test items) were, respectively, *λdif*1 = .897; *λdif*2 = .886; *λdif*3 = .914; *λdif*4 = .905; *λdif*5 = .920; *λdif*6 = .907; *λdif*7 = .911; *λdif*8 = .702. All items were associated with the latent variable at a significant level. The variance extracted for the factor was VE = .779, and reliability CR = .965.

**Representation of the child**^4^ 4The latent variable consisted of the following observable variables:r1 My child does unnecessary things.r2 My child wastes a lot of time on things that are trivial and unimportant.r3 My tasks are more important than the tasks of my child.r4 My child’s activity is of little value.r5 My goals are more important than those of my child.r6 My activity is more important than the activity of my child.r7 My ideas are more important than the ideas of my child.r8 My needs are more important than the needs of my child.consists of items belonging to the scale of the Representation of the Child. Lambda (factor loadings) of the observable variables (test items) were, respectively, *λr*1 = .920; *λr*2 = .943; *λr*3 = .802; *λr*4 = .657; *λr*5 = .880; *λr*6 = .851; *λr*7 = .616; *λr*8 = .801. All items were associated with the latent variable at a significant level. The latent variable consists of two factors. The first factor consists of questions relating to the representation of the child’s tasks as less important than those of the parent. This is shown in the wheel with the description in Fig. 2 *the child’s tasks*. The second factor contains questions relating to the representation of the child’s tasks as more important than those of the parent. It is marked as a circle with a description in Fig. 2 *parent’s tasks*. Variance extracted for the first factor (see Fig. 2 in the “tasks of the child” circle) is VE = .722, and reliability CR = .884. Variance extracted for the second latent variable (see Fig. 2 in the “parent’s tasks” circle) is VE = .633, and reliability CR = .895.

**Combating stress by pressure**^5^ 5The latent variable consisted of the following observable variables:S7 I can deal with difficulties associated with my child by applying pressure.S8 I use coercion when there are problems with my child.S9 When I’m having a difficulty with my child I force my child to surrender.was operationalized by items belonging to the Response to Stress scale, combating stress by applying pressure. Lambda (factor loadings) of the observable variables (the three test items) were as follows: *λs*7 = .837; *λs*8 = .937; *λs*9 = .891. All items were associated with the latent variable at a statistically significant level. Variance extracted was VE = .791. Construct reliability was CR = .919. Both reliability and variance extracted from the variable were very high.

**Withdrawal**^6^ 6The latent variable consisted of the following observable variables: S10 I’m tired of raising my child.S11 I retreat when it is difficult and I cannot get along with my child.S12 I avoid contact with my child when I lose strength to cope with my child.S13 I do not try (I give up) when difficulties arise in my relationship with my child.S14 The difficulties I experience in my relationship with my child make contact with my child very difficult.S15 I do not confront my child.was operationalized by items belonging to the factor of withdrawal, which belongs to the Response to Stress scale. Lambda (factor loadings) of the observable variables (the six test items) were as follows: *λs*10 = .790; *λs*11 = .782; *λs*12 = .812; *λs*13 = .762; *λs*14 = .830; *λs*15 = .593. All items were associated with the latent variable at a statistically significant level. Variance extracted is VE = .586 and reliability is CR = .893. Variance extracted of the latent variable is therefore quite good and reliability of the variable can be assessed as very good.

**Cognitive Distancing**^7^ 7The latent variable consisted of the following observable variables:S2 I can deal with bringing up my child (even if it is difficult for me).S3 When something goes wrong in my relationship with my child I know that in the end I will find a solution.S4 I am not discouraged by difficulties in bringing up my child, I can see them from a distance.was operationalized by items belonging to the factor of the cognitive distancing, which belongs to the Response to Stress scale. Lambda (factor loadings) of the observable variables (the three test items) were as follows: *λs*2 = .897; *λs*3 = .892; *λs*4 = .839. All items were associated with the latent variable at a statistically significant level. Variance extracted is VE = .768 and reliability is CR = .908. Both reliability and variance extracted for the variable are very high.

**Seeking help**^8^ 8The latent variable consisted of the following observable variables:S1 I seek solutions in difficult situations concerning my child and myself.S5 I look for specialist support when I have difficulties in bringing up my child.S6 I seek various consultations as I do not know how to bring up my child.was operationalized by items related to the seeking help of the Response to Stress scale. Lambda (factor loadings) of the observable variables (the three test items) were as follows: *λs*1 = .375; *λs*5 = .845; *λs*6 = .990. All items were associated with the latent variable at a statistically significant level. Variance extracted is VE = .612 and reliability is CR = .807. Both reliability and variance extracted are very high.

In conclusion, it should be noted that the formed latent variables showed very good reliability along with the percentage of variance extracted. Apart from the latent variable of the representation of the child, the reliability and percentage of variance extracted for the other variables had very good psychometric parameters (Table 3).

**Table 3:** Fit statistics of the measurement model.

Obtained values	Expected values in order not to reject H0
*χ*^2^(604) = 1445.683; *p* < .0005	*p* > .05
df = 604	
*χ*^2^∕df = 2.394	<2.5
CFI = .907	>.900
RMSEA = .066	<.08

The model presented here has many degrees of freedom (604). This means that it is complex (in the sense that it has many free parameters, not calculated). The value of the most important fit statistic RMSEA (<.08) indicates that the model fits the data well (Aranowska, 2005; Bartholomew et al., 2008; Hair et al., 2006; Heck & Thomas, 2009; Heck, Thomas & Tabata, 2010; Konarski, 2009; Preacher et al., 2008). The value of the test was *χ*2∕*df* = 2.394 (which is lower than the criterion of 2.5), and thus the measurement model can be considered as fitting the data well. The value of CFI, which is slightly higher than .9, also shows that the model fits the data well.

The structural equation model was calculated after calculating the measurement model and finding that it is correctly built (as indicated by the good construction of the latent variables, latent variable discriminatory power and good fit of the model to the empirical data).

## Results of Calculations of the Estimators in the One-level Structural Equation Model (SEM)

**Figure 3:**
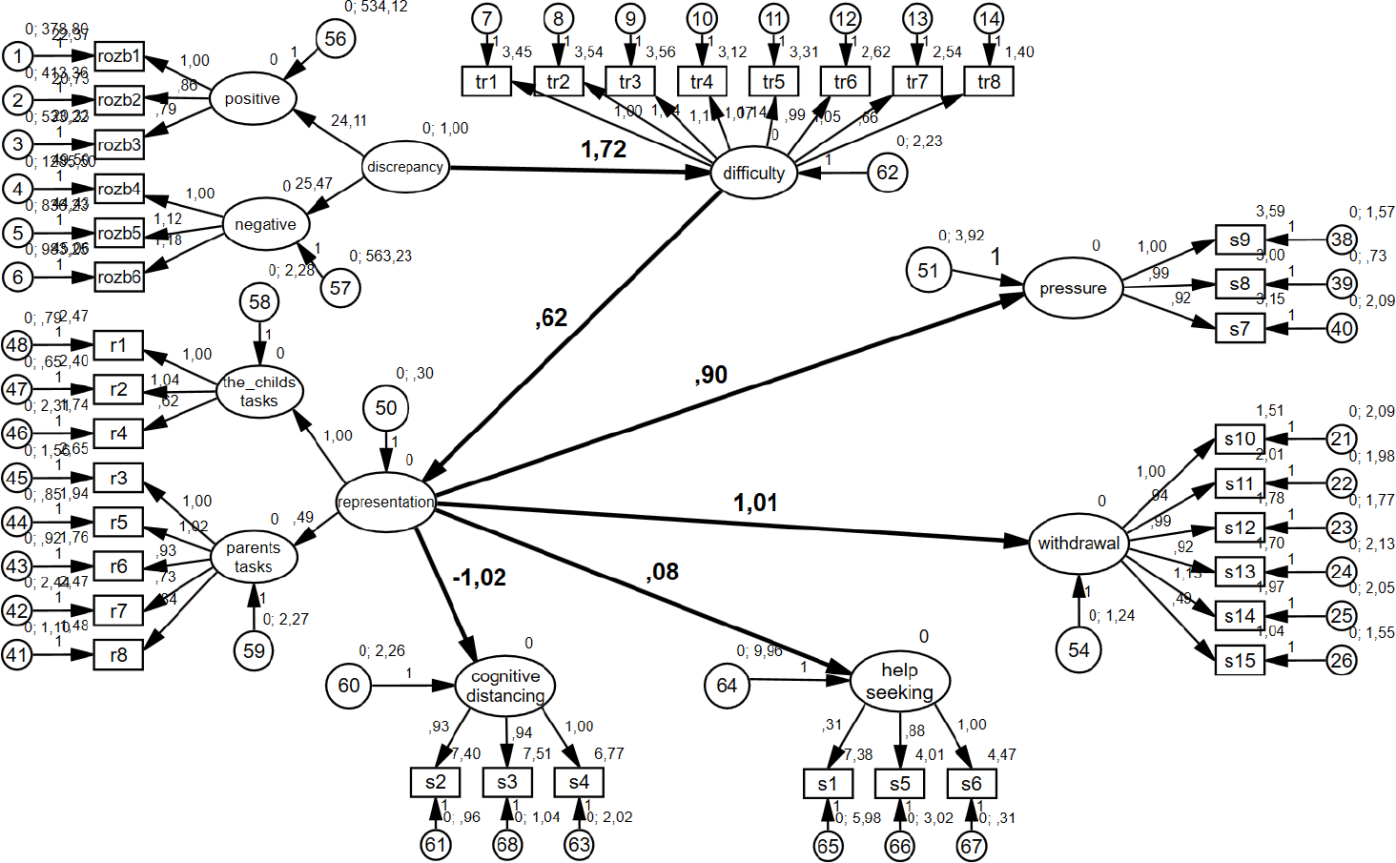
Graphical representation of the model. Non-standardized results.

The theoretical model was verified by means of structural equations modeling. Degrees of freedom were freed in such a way that only relations provided at the theoretical level were left (Fig. 1). The results of the models are shown in the graphs presenting the standardized results (Fig. 2) and non-standardized results (Fig. 3).

The measures presented in Table 4 indicate a very good fit to the data of the proposed model. In particular, the value of the RMSEA statistic (which does not exceed the critical value of .08) and the value of the CFI statistic (.905) indicate that the presented model describes the data well. In other words, there is no basis to reject the null hypothesis stating that there are no differences between the theoretical model and the empirical data.

**Table 4:** Model fit statistics.

Fit indexes	Value	Value recommended H0 for non-repudiation	Level of statistical significance
*χ*^2^	1482.983		*p* < .001
df	619		
*n*	319		
*χ*^2^*independent*	9759,294		*p* < .001
df *independent*	703		
*χ*^2^/df	2.396		
CFI	.905	>.900	
RMSEA	.066	< .06 < .08	90% probability

The **first hypothesis** postulated that the discrepancies of the parental goals to the child’s current level of development were positively associated with the difficulty experienced by the parent. The path between the discrepancy and the experienced difficulties is *γ*_11_ = .75; *p* < .0005 (Fig. 2), (*γ* = .53, *p* < .005 in earlier study: (Szymańska, 2011b)).

As can be observed, the discrepancy determines the difficulty experienced by the parent. The regression determinant for this relation is 1.72, which means that when the discrepancy increases by one unit, the difficulty experienced by the parent increases by 1.72 units (Fig.  3). The discrepancy variable explains 56.2% of the variability of the experienced difficulties variable [(.75)2 = .562]. This is a significant value.

Colloquially speaking, the phenomenon here is that the child “does not live up to the parent’s expectations”. The parent wants the child to be characterized by other features but the child has developed them to an extent that is not good enough, or the child has developed other personality traits that the parent does not desire.

The **second hypothesis** postulated that the difficulty experienced by the parent is positively associated with the formation of the representation of the child in the parent’s mind. The path between the construct of the difficulties experienced by the parent and the child’s representation in the parent’s mind is *β*_21_ = .930; *p* < .0005 (Fig. 2) (*β* = .69, *p* < .005 in earlier study: (Szymańska, 2011b)). The regression determinant is .62, which means that when the difficulty experienced by the parent increases by one unit, the child’s representation increases by .62 units (Fig. 3). The variable of the experienced parental difficulty explains 86.5% of the variability of the variable of the child’s representation [(.93)2 = .865].

As a result of experiencing difficulties in the parenting situation resulting from the inability to fulfill the parent’s objectives, a specific representation can be built of a child as a person whose objectives and activities are less important than the objectives and activities of the parents. The child in such a situation can be considered the person who has less to say in the mutual relationship than the parent and the one who should follow the direction marked out by that parent.

The **third hypothesis** postulated that the child’s representation in the parent’s mind determines combating stress by applying pressure. The relationship between the constructs proved to be statistically significant. The path was *β*_62_ = .570; *p* < .0005 (Fig. 2). This result supports the validity of the proposed hypothesis—the relationship between the constructs is positive and moderate. The variable of representation explains 32.5% of the variability of the variable of combating stress by applying pressure (.570)2 = .325. The regression coefficient for this relationship is .90, which means that when the child’s representation increases by one unit, combating stress by applying pressure increases by .90 units (Fig. 3).

The **fourth hypothesis** postulated that the representation of the child in the parent’s mind determines the defense reaction against stress by withdrawal. The relationship between the constructs proved to be statistically significant. The relationship was *β*_32_ = .800; *p* < .0005 (Fig. 2), (*β* = .60, *p* < .005 in earlier study: (Szymańska, 2011b)). This result supports the hypothesis that the relationship between the constructs is positive and high. The variable of the representation explains 64% of the variability of the variable defense against stress, (.800)2 = .640. The regression coefficient for this path is 1.01, which means that when the child’s representation increases by one unit, defense against stress increases by 1.01 units (Fig. 3).

Parents experiencing difficulties as a result of the inability to achieve their objectives may withdraw from the parenting situation. This difficult situation for the parent can make that parent start to avoid interaction with the child, trying not to lead to confrontation and, as a result, withdrawing from the parenting situation.

The **fifth hypothesis** postulated that the child’s representation is associated with the variable of seeking help (Fig. 1). The relationship between the constructs proved to be irrelevant. There is no relationship between the representation of the child in the parent’s mind and the parent’s seeking help.

In case of difficulties as experienced by the parent, the parent can try to solve the problem by trying to seek support, or the parent can obtain advice from the child’s grandmother, grandfather or from a psychologist, etc. It would seem that parents would naturally have the ability to reach for this type of assistance.^9^ 9In Poland, where the research was carried out, a large number of psychologists work in preschools. Each preschool is also under the care of a pedagogical-psychological clinic.

The **sixth hypothesis** postulated that the representation of the child in the parent’s mind is associated with the variable of combating stress by cognitively distancing oneself. In this case the significance of the relationship between the constructs was also confirmed *β*_52_ =  − .710; *p* < .0005 (Fig. 2), (*β* =  − .72, *p* < .005 in earlier study: Szymańska, 2011b). This supports the validity of the sixth hypothesis. It revealed that the relationship between the constructs is negative and high. The child’s representation variable explains 50.4% of the variability of the variable of combating stress by distancing oneself (−.710)2 = .504. The regression coefficient for this path is −1.02, which means that when the representation of the child increases by one unit, combating stress by distancing oneself decreases by −1.02 units (Fig. 3).

A parent who distances him or herself from the situation maintains a reserved, cool assessment of the situation and takes steps to resolve the problem. The parent observes the situation from different perspectives and selects steps that could help to solve the problem. It would seem that this is one of the most reasonable ways of coping with a difficult situation. It is also a strategy which is considered to be one of the possible ways of dealing with stress. This would be a highly desirable response in the situation of difficulties experienced by the parent.

From the results obtained here, it must be concluded that the model fits the data well, which confirms the validity of the theoretical model. All the relations in the model, except representation of the child in the parent’s mind and searching for help, are significant. Four model relations were high (higher than .700) and one was moderate (.570).

It must be stated that as a result of the formation of the representation of the child and the fact that the child’s tasks are less important than the activities of the parent, the parent adopts combating the stress reaction by applying pressure to the child and defends against stress by withdrawal. However, parents do not adopt combating stress reactions which involve searching for help or distancing themselves from the situation.

## Discussion

The theoretical model was verified in the study. It proved to be well matched to the data, thus confirming the correctness of the theoretical model. The model describes six relationships between its elements.

The study demonstrated that discrepancy and inability to achieve parenting goals in the group of parents of preschool children is strongly associated with the parent’s experiencing difficulties in their relationship with the child.

The hypothesized relationship between the difficulty experienced by the parent and the child’s specific representation in the parent’s mind (involving perception of the child and that child’s activities as less important than the goals, objectives and activities of the parent) was confirmed. The study revealed that the relationship is very high (*β* = .93). It is therefore clear that experiencing parenting difficulties arising from the inability to achieve objectives is very strongly associated with the representation of the child and with that child’s activity as a person of less importance.

As a result of experiencing parenting difficulties, parents tend to react in a specific way depending largely on their personal qualities, temperament and style of coping with stress. Four hypotheses concerning four styles of coping with experienced difficulty in the parenting situation were put forward. These styles were: applying pressure, withdrawal, seeking help, and distancing oneself.

Parental application of pressure to the child is moderately positively related to parental experience of difficulties.

The relationship between the representation of the child in the parent’s mind and withdrawal from the parenting situation is strong *β* = .80. When the parent is experiencing difficulties arising from the inability to fulfill his/her parenting objectives, he/she withdraws from the situation. This may impair interaction with the child, and the child might not understand why the parent is not talking to him or her. The parent’s withdrawal from the interaction can be understood by the child as winning (the parent surrenders) or, on the contrary, as the parent’s reluctance or abandonment of the child.

The relationship between the representation of the child in the parent’s mind and seeking help, as shown by the results, is not correlated. It is difficult to estimate the possible consequences on the child’s development by defecting from the difficult situation and leaving the child unassisted.

The relationship between the representation of the child in the parent’s mind and the parent’s cognitive distancing him or herself from the parenting situation is strong but negative (*β* =  − .71). It cannot therefore be expected that a parent will distance him or herself from the situation of experiencing parenting difficulties resulting from the inability to achieve his/her goals.

The results reveal that parents who experience difficulties in the parenting situation are most likely to respond by withdrawal from the parenting situation or to apply pressure. The study revealed that experiencing difficulties in the parenting situation is not associated with help-seeking by the parents. It is also negatively associated with cognitive distancing oneself from the situation.

The two most desirable reactions in situations of experienced difficulties are practically not employed. The study was designed so as to obtain information about the likely reaction of the parents to the situation of experienced parenting difficulties. The study did not explain the other reasons for the application of these reactions. Seeking help and cognitive distancing oneself from the situation in which the parent is experiencing difficulties could help him or her to find a solution and perhaps prevent escalation of the conflict with the child.

The results are surprising, especially considering the fairly common knowledge of how to deal with parenting difficulties (workshops on this topic are conducted in Polish preschools, and psychological help is also available). The parents’ declarations clearly reveal that when experiencing difficulties they have the most basic and elementary responses: coping with stress by escaping or by using pressure (Selye, 1956).

The results may suggest that despite the possibility of using different forms of coping with difficulties, the specificity of the difficulties experienced in the parenting process in some way makes it more difficult for parents to deal with stress more constructively.

The educator Janusz Korczak wrote: *in this sense of being lost you can easily become a tyrant when you do not realize that when you act in a difficult situation you can make a mistake, an unrecoverable mistake* (Gurycka, 2002). Gurycka too pointed out the strong association between coping with stress by applying pressure and the development of parental mistakes. Hence, frequent experiencing of difficulties was associated in her theories with the emergence of *parental mistakes* which, in turn, create the risk of negative consequences for the child’s development (Gurycka, 1990; Gurycka, 2008). Coping with stress can therefore be an intermediate variable in the formation of parental mistakes.

## Conclusions

The study was carried out on a fairly large sample of more than 300 participants from all over Poland. The sample has many features of a representative sample, as indicated by the demographic variables, such as place of residence or education. Unfortunately, in our sample fathers’ are grossly underrepresented. Results for the male population should be interpreted very carefully. Although Gurycka’s model is universal (Gurycka did not limit it to any age group), in order to confirm the model for the preschool age population, we decided to test it on a group of preschool children’s parents. This was a research limitation.

The data were obtained through self-report because some of the variables were very subjective and could only be accessed by asking the person. These variables were: discrepancy, experiencing difficulties or representation of the child. Likewise, the response to stress variables could only be tested by self-report. It should be noted, however, that this kind of data limits the validity of the findings.

The study was cross-sectional, not longitudinal. This has implications for the SEM models. Only longitudinal studies enable maintenance of dependencies in SEM models in terms of direct or indirect causality (Hair et al., 2006; Heck & Thomas, 2009). Therefore, the present model is descriptive rather than cause–effect. It describes the relationships between the variables specified in the hypotheses. The theoretical model described by Gurycka describes these relationships in cause–effect terms (Gurycka, 1990).

Another important methodological issue is the construction of alternative models. The phenomenon of parents’ reaction to stress we described here can be explained by a number of variables. These include the child’s temperament, the parent’s personality traits, the socio-economic status of the family, and many others which have already been described in the literature (Oddi, Murdock & Vadnais, 2013; Casalin et al., 2014).

The model we reconstructed on the basis of Gurycka’s theory is unique because the variable of discrepancy of parental goals and child’s characteristics is presented as the cause of parental experienced difficulties, which consequently shape the representation of the child in the parent’s mind and the parent’s response to stress. This new approach has not been tested before. Therefore this model, and no other alternative models, was tested.

It should also be added that we do not know what the alternative model to the one presented here is, one that would present the relationships between the variables in a different way: discrepancies, parental difficulties, representation of the child and stress response. Such a model could be generated empirically but its status would still be smaller than the theoretical model presented here (Hair et al., 2006; Szymańska, 2016).

The present model confirms the following relations found in an earlier study of a different Polish samples (Szymańska, 2011b):

1. between discrepancy and experienced difficulty *β* = .53, *p* < .005 (earlier study) and *β* = .75, *p* < .005 (present study);
2. between difficulty and representation of the child *β* = .69, *p* < .005 (earlier study) and *β* = .93, *p* < .005 (present study);
3. between representation of the child and withdrawal *β* = .60, *p* < .005 (earlier study) and *β* = .80, *p* < .005 (present study);
4. between representation of the child and cognitive distancing *β* =  − .72, *p* < .005 (earlier study) and *β* =  − .72, *p* < .005 (present study).

The present model has been extended compared with the earlier model. Two new variables were tested in the present study: pressure and help seeking.

However, caution must be exercised when generalizing the presented findings to populations from other countries. Relations between variables in other countries may be similar to the Polish ones but they may well be stronger or weaker depending on the magnitude of the discrepancy experienced by parents, i.e., on the first variable in the model. This discrepancy may be culturally determined. In countries where children go to school earlier, or where the educational system demands greater discipline, expectations that children will develop certain competencies such as responsibility, truthfulness or patience may be much greater. Parents may therefore be expected to make sure that their children develop these competencies. This is what happens in Poland. Parents are trained to prepare their children to go to school. If their children are unable to meet these expectations i.e., develop these competencies, their parents will probably experience discrepancy and the processes described in the presented model will be triggered.

One must remember that parental goals depend on prevalent cultural norms and values and therefore they will surely affect the upbringing process (LeVine, 1974; LeVine, 1980; Kuczyński, 2003; Szymańska & Aranowska, 2016). Hence the relations in our model may be strongly culturally determined. Of course the presented example is just one of many possible examples of how cultural norms may affect the modelled relations. We know about these possible effects thanks to the work on relations between parenting goals and cultural norms (LeVine, 1974; LeVine, 1980). These cultural effects definitely deserve further consideration.

##  Supplemental Information

10.7717/peerj.3384/supp-1 Supplemental Information 1 Data set in SPSS which contains all variables considered in the analysis Variables in the data set: parent_age, parent_sex, residence, education_level; Variables belonging to the variable DIFFICULTY: tr1, tr2, tr3, tr4, tr5, tr6, tr7, tr8; Variables belonging to the variable REPRESENTATION: r1, r2, r3, r4, r5, r6, r7, r8, child_age, child_sex, kindergarten_type; Variables belonging to the variable DISTANCING: s2, s3, s4; Variables belonging to the variable HELP SEEKING: S1, s5, s6; Variables belonging to the variable PRESSURE: s7, s8, s9; Variables belonging to the variable WITHDRAWAL: s10, s11, s12, s13, s14, s15; Variables belonging to the variable DISCREPANCY: rozb1, rozb2, rozb3, rozb4, rozb5, rozb6

10.7717/peerj.3384/supp-2 Supplemental Information 2 How to connect “BASE_FOR_REVIEW_STRESS.SAV” to AMOS program in which the results were calculated

10.7717/peerj.3384/supp-3 Supplemental Information 3 CFA model in AMOS

10.7717/peerj.3384/supp-4 Supplemental Information 4 SEM model in AMOS

## Additional Information and Declarations

### Data Availability

The following information was supplied regarding data availability: The raw data has been supplied as a Supplemental Dataset.
