# Supplementary material for: The ways parents cope with stress in difficult parenting situations: the structural equation modeling approach
Source: PeerJ. 2017 Jun 12;5:e3384. doi: 10.7717/peerj.3384 (PMC5470577; doi:10.7717/peerj.3384)

I send additional information for data set.

I send graphs of CFA model and SEM model in AMOS program for reviewers convenience.

The results were calculated in AMOS 22 (this is important as other versions differ slightly in estimations for example: 0,722 instead of 0,723). Maximum Likelihood estymator was used.

In order to calculate, please, click:

File – Data Set – File name (select file) – OK.

Analyze – calculate estimates

If You would like to see numbers in the graph please click red arrow.


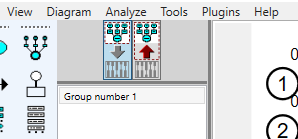


If You want standardized estimates please click this option.


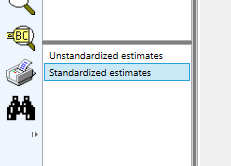


To see whole results

View – text output

And you will see this


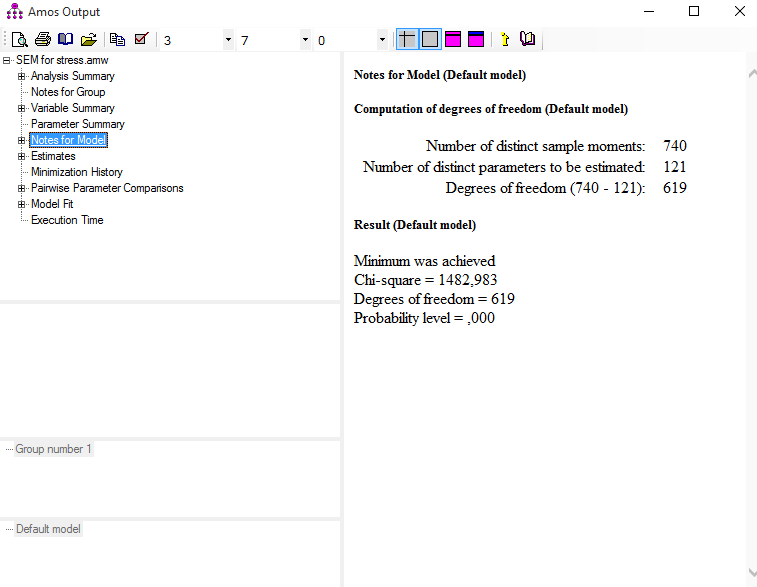

Supplement: Supplemental Information 2 [file peerj-05-3384-s002.docx]
